# A Novel Single Base Mutation in *OsSPL42* Leads to the Formation of Leaf Lesions in Rice

**DOI:** 10.3390/ijms252211871

**Published:** 2024-11-05

**Authors:** Panpan Li, Huihui Shang, Xia Xu, Junyi Gong, Jian-Li Wu, Xiaobo Zhang

**Affiliations:** State Key Laboratory of Rice Biology, China National Rice Research Institute, Hangzhou 310006, China; li_pp1203@163.com (P.L.); huihuishengji1101@163.com (H.S.); mailxuxia@163.com (X.X.); junyigong8107@163.com (J.G.); beishangd@163.com (J.-L.W.)

**Keywords:** rice, spotted-leaf mutant, map-based cloning, bacterial blight, lignin, defensive response

## Abstract

Rice spotted-leaf mutants serve as valuable resources for studying plant programmed cell death (PCD) and disease resistance mechanisms, making them crucial for research on disease resistance in rice. Map-based cloning was used to identify and clone the spotted-leaf gene *OsSPL42*. Then, functional complementation and CRISPR/Cas9 techniques were also employed to further validate the function of this gene. By applying leaf clippings for bacterial blight (BB) inoculation, the BB resistance of different rice lines was assessed. The results in this study were as follows: The *OsSPL42* behaved as a recessive nuclear gene and was narrowed down to a 111 kb region on chromosome 8. All T_0_ transgenic rice plants in the complementation experiments exhibited a wild-type phenotype, without any lesion spots on the rice leaves. This suggests that the *LOC_Os08g06100* encoding O-methyltransferase is the candidate gene for the mutant *spl42*. The *OsSpl42* is widely expressed and the OsSPL42-GFP protein is mainly localized in the cytoplasm. *OsSPL42* overexpression lines are more susceptible to BBs, which indicates that *OsSPL42* may act as a negative regulator of rice resistance to BB. In summary, we speculate that *OsSPL42* plays an important role in the regulation of pathogen response, providing new insights into plant defense mechanisms.

## 1. Introduction

The formation of leaf lesions that mimic the hypersensitive response (HR) is an abnormal phenomenon due to variations in internal genetic factors or environmental stresses in plants [1]. In rice, these mutants are typically referred to as spotted-leaf mutants, which are valuable models for studying chlorophyll metabolism, phytohormone signaling, reactive oxygen species (ROS) homeostasis, programmed cell death (PCD), and innate immunity [2,3,4].

The formation of spotted leaves in rice is largely associated with chlorophyll metabolism [5]. For instance, genes related to chlorophyll synthesis, such as *OsCHLH*, *OsPORB*, and *OsCAO1*, are key genes affecting chlorophyll synthesis [6,7,8]. The *OsCHLH* gene encodes a subunit of magnesium ion chelatase (Mg-chelatase), which is the first step in chlorophyll synthesis. Mg-chelatase is responsible for embedding magnesium ions (Mg^2+^) into precursor molecules [5]. Mutations or deletions in *OsCHLH* result in the inability to successfully embed magnesium ions, leading to limited chlorophyll synthesis. This not only results in a drop in chlorophyll content but also triggers uneven pigmentation of the leaves, eventually forming spots. *OsPORB* encodes a subtype of protochlorophyllide oxidoreductase (POR), a light-dependent precursor of chlorophyllide. Under light conditions, the enzyme converts the protochlorophyllide precursor into chlorophyll. Mutations or downregulation of *OsPORB* expression lead to an incomplete step, resulting in a decrease in chlorophyll content and the appearance of blotches [9]. *OsCAO1* (chlorophyllide a oxygenase 1) is involved in the conversion of chlorophyllide b into chlorophyllide a. This step is critical for the stability of chlorophyll molecules in the chloroplast complex [6]. Mutation or loss of *OsCAO1* results in reduced chlorophyll stability, which may lead to the formation of spotted leaves.

In addition to genes related to chlorophyll synthesis and degradation, the spot formation in rice spotted-leaf mutants is also related to the plant hormone signaling pathway. Notably, plant hormones such as gibberellin (GA), indole acetic acid (IAA), and jasmonic acid (JA) play an important role in this process. The physiological and biochemical changes of the formation mechanism of rice mutant spotted leaves include many aspects involving gene expression and regulation, as well as a series of biochemical pathways and physiological processes [10].

The formation of spotted leaves is usually accompanied by the accumulation of ROS, leading to an imbalance in the REDOX reaction, and then causing a series of physiological and biochemical changes. Excessive ROS can cause PCD and HR [11]. To maintain the balance of ROS, multiple ROS scavenging enzymes are present in plants, such as catalase (CAT), superoxide dismutase (SOD), peroxidase (POD), ascorbate peroxidase (APX) and other enzymes [12]. Studies have shown that the leaf tissue of spotted-leaf mutants undergoes PCD mediated by genetic programming [13]. This phenomenon usually causes mutant plants to have high disease resistance, thus preventing the invasion and spread of pathogens in the environment [14,15,16,17,18,19,20]. Some studies have shown that PCD plays a certain role in preventing the invasion and spread of rice blast and BB. The spotted-leaf mutants generally showed increased resistance to fungi and bacteria [21]. This change makes spotted-leaf mutants an important resource for studying the innate immune mechanism of plants. Therefore, the identification and study of spotted-leaf mutants are of great significance for the analysis of the mechanism of action of spotted-leaf mutants and the innate immune mechanism of plants.

Rice spotted-leaf mutants are extensively studied due to their enhanced disease resistance against a range of pathogens, particularly BB caused by *Xanthomonas oryzae* pv. *Oryzae (Xoo)*. Notable mutants such as *spl11* [22], *spl30* [23], *spl24* [24], *spl40* [25], *Spl26* [26], and *spl41* [27] exhibit significant resistance to BB. *spl24* is a novel lesion mimic mutant with semi-dominant inheritance. This mutant enhances innate immunity and facilitates the identification of key genes involved in disease resistance, which can be applied in breeding strategies [24]. Similarly, *spl40* has been shown to confer strong resistance to BB by modulating the jasmonic acid (JA) and salicylic acid (SA) signaling pathways. The upregulation of these pathways in *spl40* triggers systemic acquired resistance (SAR), which is crucial for its defense response [25]. *spl11*, originally derived from mutagenized rice populations, shows non-specific resistance to both rice blast and BB, possibly through a broader defense activation mechanism. The non-specific nature of this resistance points to the potential involvement of generalized immune pathways rather than pathogen-specific responses [22]. However, despite forming leaf lesions, *spl30* does not exhibit typical signs of PCD. This unusual phenotype is hypothesized to result from the accumulation of as yet unidentified pigments rather than the activation of cell death-related pathways. The absence of PCD in *spl30* distinguishes it from other lesion mimic mutants and suggests an alternative resistance mechanism that may not rely on cell death for defense [23]. The *Spl26* gain-of-function mutation positively regulates plant immunity by enhancing ROS production and promoting cell death, which is central to its heightened resistance to BB [26]. Conversely, the *spl41* mutant acts as a negative regulator of plant immunity. This mutation impairs defense responses, providing insights into the fine-tuning of immune pathways and the potential for manipulating such genes in breeding programs [27]. These mutants have been incorporated into rice breeding programs, utilizing their enhanced resistance traits to improve the overall disease resistance of commercial varieties. The spotted-leaf mutants, particularly those like *spl24* and *spl40*, which activate key immune pathways, have been instrumental in creating rice lines with durable resistance. These mutants were originally identified through techniques such as EMS mutagenesis, and their integration into breeding schemes has led to the development of rice cultivars with robust, field-level resistance to BB. The mechanism of resistance in most of these mutants can be traced back to the spontaneous induction of programmed cell death, which plays a central role in limiting pathogen spread (except for *spl30*). Future studies should focus on environmental factors influencing the expression of resistance traits in these mutants. Understanding the environmental plasticity of their resistance mechanisms will be essential for optimizing their deployment in different agroecological zones. Such research will contribute to the enhancement of commercial rice varieties, ensuring greater adaptability and resilience in the face of pathogen pressure and changing climatic conditions.

In this study, we isolated a novel rice spotted-leaf mutant *spl42* from an ethyl methyl sulfonate (EMS)-induced mutant bank of the indica variety of ZJ100. A point mutation in *OsSPL42* was identified and was responsible for the lesion formation of *spl42*. *OsSPL42* was widely distributed and localized to cytoplasm and might negatively regulate disease resistance to *Xoo* in rice.

## 2. Results

### 2.1. OsSPL42 Controls the Spotted-Leaf Phenotype of spl42

**Figure 1:**
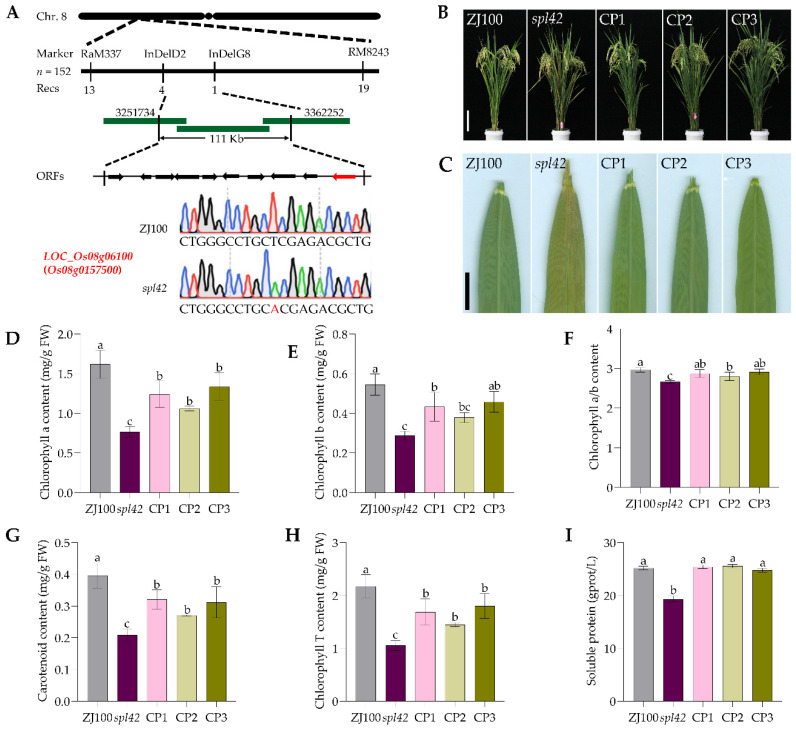
Identification and functional validation of *OsSPL42*. (**A**) The red letter A represents the mutation site, including T directly above A, showing the wild-type ZJ100 mutating from T to A. (**B**) Plant phenotype of wild-type ZJ100 and mutant *spl42*, and complementary lines at the filling stage. Bar = 17 cm. (**C**) Top second leaves of phenotype of wild-type ZJ100 and mutant *spl42*, and complementary lines at the tillering stage. Bar = 1 cm. (**D**) Chlorophyll a content. (**E**) Chlorophyll b content. (**F**) The ratio of chlorophyll a to chlorophyll b. (**G**) Carotenoid content. (**H**) Total chlorophyll content. (**I**) Soluble protein. CP, complementation; values are means ± SD, *n* = 3; and different letters indicate significant differences by one-way ANOVA and Duncan’s test *p* ≤ 0.05.

In this study, the mutant *spl42* was obtained through EMS mutagenesis of ZJ100, and it was determined that the spotted-leaf phenotype of the mutant is controlled by a single recessive gene. At the same time, approximately 200 pairs of SSR markers and 10 pairs of InDel markers were used to map the candidate gene. The candidate gene was narrowed down to a 111 kb region between InDelD2 and InDelG8 (Table S1). Using the website Gramene (http://www.gramene.org/, accessed on 8 March 2021), we found ten open reading frames (ORFs) in the 111 kb region. The DNA sequences of ten ORFs were PCR amplified and sequenced, and then the DNA sequence from *spl42* and the wild control were carefully aligned. The results showed (Figure 1A) that the ORF10, *LOC_Os08g06100*, had a single base substitution (T247A) at the 247th base of the gene, resulting in the change of the 42nd amino acid (AA) (L42H). Therefore, the *LOC_Os08g06100*, which encodes O-methyltransferase involved in lignin monomer biosynthesis [28], was supposed to be the candidate gene.

To further determine whether the mutation of this gene leads to the growth and development defects of the mutant, we conducted functional complementation experiments. The binary vector pCAMBIA1300-OsSPL42 containing the *OsSPL42* promoter and genomic coding region was introduced into the mutant *spl42* callus. We obtained 16 positive transgenic plants confirmed by PCR assistant DNA sequencing. All the 16 *spl42* genetic background transgenic plants showed no lesion mimic phenotype as *spl42*. We selected three of them to collect plenty of seeds and they were named lines CP1, CP2, and CP3 for the next experiments (Figure 1B,C). By observing the phenotype of T_1_, it was found that the T_1_ complementary transgenic lines showed phenotypic separation, and all plants exhibiting a wild-type phenotype were transgenic positive. This indicated that the single T_0_ positive transgenic strain was heterozygous, confirming that *OsSPL42* is successfully complementary to the mutant *spl42*, and further proving that *OsSPL42* is a single recessive gene controlling spotted leaves.

In addition, photosynthetic pigment and soluble protein contents in CP1, CP2, CP3, *spl42*, and wild-type were detected (Figure 1D–I). The results showed that the contents of chlorophyll a, chlorophyll b, chlorophyll a/b, carotenoids, total chlorophyll, and soluble protein in CP1, CP2, and CP3 were significantly higher compared to mutants. The levels of photosynthetic pigments and soluble protein content in the three complementary lines were partial recovery. CP1, CP2, and CP3 only recovered to 76.7%, 65.5%, and 82.6% of the ZJ100 of the chlorophyll a content, respectively, showing partial recovery but not a full recovery to the wild-type level. However, the chlorophyll a content of *spl42* recovered to 47.3% of ZJ100, which was significantly lower than the complementary lines. This is a very common phenomenon. The number of chloroplasts in living cells showed partial recovery, and necrotic spots did not appear when *OsSPL42* was introduced into the mutant. It was speculated that the appearance of spotted leaves during the growth and development of the mutant was caused by a mutation in the *OsSPL42* gene.

Agronomic traits of ZJ100, *spl42*, and three complementary lines were investigated (Table 1). Most agronomic traits were also recovered after functional complementation, indicating that the growth and development of the mutant was caused by a mutation in *OsSPL42*, resulting in alterations in agronomic traits. However, the 1000-grain weight of the complementary lines was lower than that of both mutant wild types. This indicates that the lower 1000-grain in *spl42* is not caused by the mutation in *OsSPL42*.

**Figure 2:**
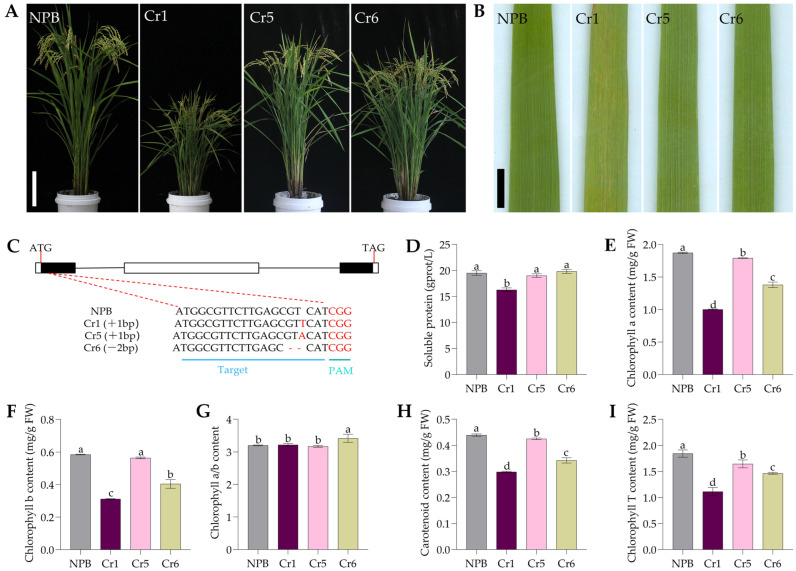
Phenotypic identification and functional verification of *OsSPL42* knockout strain. (**A**) Plant phenotype of NPB and knockout line at filling stage. Bar = 17 cm. (**B**) Leaves of phenotype of NPB and knockout line at filling stage. Bar = 1 cm. (**C**) Knockout target sequence. (**D**) Soluble protein. (**E**) Chlorophyll a content. (**F**) Chlorophyll b content. (**G**) The ratio of chlorophyll a to chlorophyll b. (**H**) Carotenoid content. (**I**) Total chlorophyll content. Cr, knockout; values are means ± SD, *n* = 3; and different letters indicate significant differences by one-way ANOVA and Duncan’s test *p* ≤ 0.05.

We also utilized CRISPR/cas9 technology for gene editing of *OsSPL42* in Nipponbare (NPB) callus. A total of 19 *OsSPL42* knockout lines were obtained. None of these 19 knockout lines showed leaf lesions in T_0_. Only one homozygous knockout line (Cr1) in the T_1_ generation exhibited leaf lesions. This line and the two other 2 homozygous knockout lines (Cr5 and Cr6) were selected for the next experiments (Figure 2) to explore the function of *OsSPL42* gene. The accumulation of soluble proteins and chlorophyll was detected in the wild-type NPB and three knockout lines. The results showed that the levels of chlorophyll a, chlorophyll b, chlorophyll a/b, carotenoids, total chlorophyll, and soluble proteins in Cr1 were significantly lower compared to the wild type. In contrast, the contents of Cr5 and Cr6 did not show significant differences compared to the wild type.

### 2.2. Leucine in OsSPL42 Is Highly Conserved

**Figure 3:**
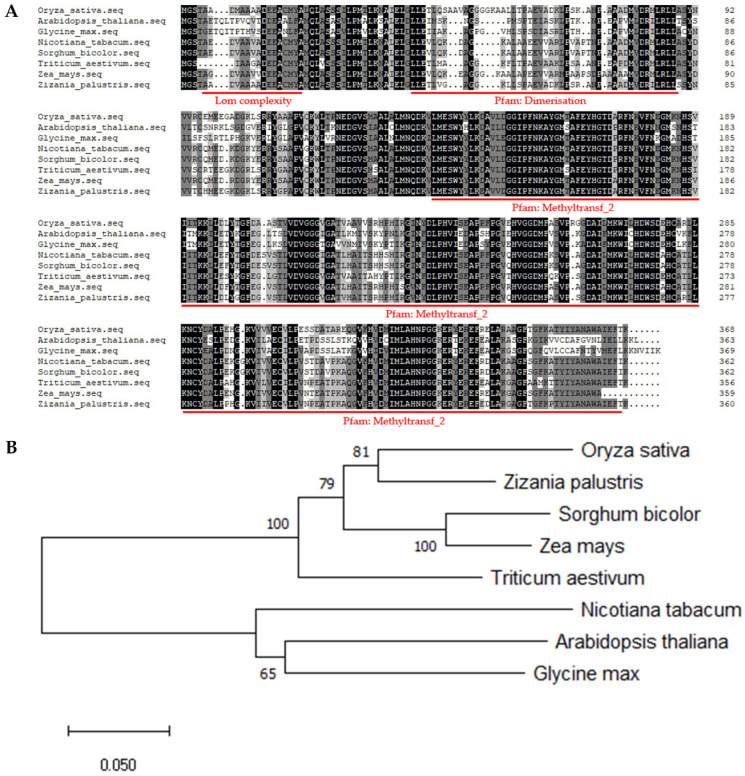
Homologous sequence alignment and evolutionary tree analysis of OsSPL42. (**A**) OsSPL42 homologous sequences were compared by DNAMAN v7 software (part), and the part underlined in red represents the domain; (**B**) construction of an evolutionary tree using the adjacency method through MEGA v6 software. All SPL42 proteins are numbered in NCBI as follows: *Oryza* OsSPL42 (KAB8107446.1), *Zizania palustris*. (KAG8086825.1), *Sorghum bicolor* SbSPL42 (AFO69477.1), *Zea* ZmSPL42 (AAQ24342.1), *Triticum aestivum* TaSPL42 (NP_001392772.1), Arabidopsis *thaliana* AtSPL42 (OAO95542.1), *Glycine* GmSPL42 (KAH1255570.1), and *Nicotiana tabacum* NtSPL42 (CAA52462.1).

The CDS of *OsSPL42* gene has a total length of 1107 bases and encodes 368 AAs. The gene was predicted to contain three domains, lom complexity, dimerization, and O-methyltransferase, using http://smart.embl-heidelberg.de/ (accessed on 2 April 2021) (Figure 3A). lom complexity is a region with low sequence complexity, spanning from the 5th AA to the 20th AA. The dimerization domain is located at the N-terminus of many plant O-methyltransferases and has been shown to mediate the dimerization of several proteins. The O-methyltransferase domain includes a series of O-methyltransferases, some of which utilize S-adenosylmethionine as a substrate. The primary role of DNA methylation in prokaryotes is to protect host DNA from degradation by restriction enzymes.

Through http://www.ncbi.nlm.nih.gov/ (accessed on 18 November 2022), we performed OsSPL42 protein sequence alignment (Figure 3B). OsSPL42 orthologues were selected from four monocot plants and three dicotyledonous plants. Monocot plants besides rice includes *palustris*, *Zea may*, *bicolor*, and *Triticum aestivum*, and the accession numbers of their orthologues in NCBI are as follows, respectively: KAG8086825.1, AAQ24342.1, AFO69477.1, and NP_001392772.1. The accession numbers of the orthologs of *Arabidopsis thaliana*, *Nicotiana tabacum*, and *Glycine max* are OAO95542.1, CAA52462.1, and KAH1255570.1 in NCBI. The similarity between OsSPL42 and its orthologs varies, ranging from 59.13% in *soybean bicolor* to 87.23% in wild rice. The mutation site of the *OsSPL42* gene is in the second domain, and the mutation site was leucine in all the orthologs of these seven species. The AAs after mutation are consistent, indicating that the mutation site is highly conserved.

At the same time, we conducted an evolutionary tree analysis on these species. The results indicated that the OsSPL42 protein of rice and its homologous protein in *Zizania palustris* were located on the same branch of the evolutionary tree, indicating their close genetic relationship. Additionally, four monocotyledonous plants were clustered on one branch, while three dicotyledonous plants were grouped on another branch. The results indicated that the OsSPL42 protein is closely related to monocotyledonous plants, and its sequence is conserved.

### 2.3. Altered Physiological/Biochemical Reactions Related to ROS Scavenging System in Spotted-Leaf Mutant spl42

**Figure 4:**
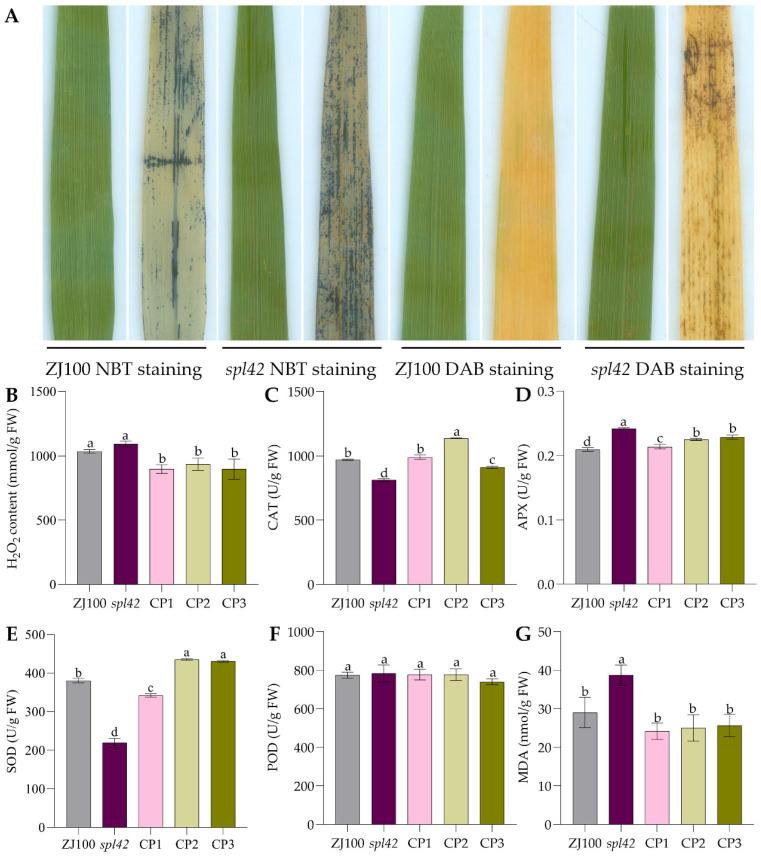
Determination of ROS accumulation of complementation lines of the *spl42* mutant at 10 weeks of age. (**A**) Wild-type ZJ100 and mutant *spl42* NBT and DAB staining (**B**) H_2_O_2_ content. (**C**–**F**) ROS scavenging enzyme activities (**G**) MDA content CP, complementation; values are means ± SD, *n* = 3; and different letters indicate significant differences by one-way ANOVA and Duncan’s test *p* ≤ 0.05.

The formation of speckled leaf spots is mostly related to ROS accumulation. To investigate whether the cause of speckled leaf spots is related to ROS accumulation, 3,3′-diaminobenzene (DAB) and nitroblue tetrazolium (NBT) staining experiments were used. The accumulation of hydrogen peroxide (H_2_O_2_) and superoxide anion (O_2_^−^) in leaves was detected. The results of the DAB staining (Figure 4A) showed obvious reddish-brown material deposits in and around the lesion site of *spl42* leaves, but not in ZJ100. There were blue material deposits in and around the lesion site of *spl42* leaves. This indicates the presence of large deposits of H_2_O_2_ and O_2_^−^ near *spl42* spots.

To further verify this result, the H_2_O_2_ content in mutant, wild-type, and complementary lines CP1, CP2, and CP3 was quantitatively determined (Figure 4B). It was observed that the H_2_O_2_ content of CP1, CP2, and CP3 was significantly lower than that of the mutant *spl42*. Additionally, the H_2_O_2_ content of ZJ100 was lower than that of the mutant, although it did not reach a statistically significant level. However, the trend was basically consistent with that of CP1, CP2, and CP3, all of which were lower than that of the mutants. The CAT activity of CP1, CP2, CP3, and ZJ100 plants was significantly higher than that of the mutant (Figure 4C). This indicates that the activity of the H_2_O_2_ scavenging enzyme was decreased in the mutant plants, resulting in the accumulation of H_2_O_2_, which caused cell death at the leaf spot. However, when the *OsSPL42* gene was introduced into this mutant, H_2_O_2_ deposition disappeared, and CAT activity recovered. This suggests that the *OsSPL42* gene might regulate the ROS outbreak in rice plants (Figure 4D–G).

**Figure 5:**
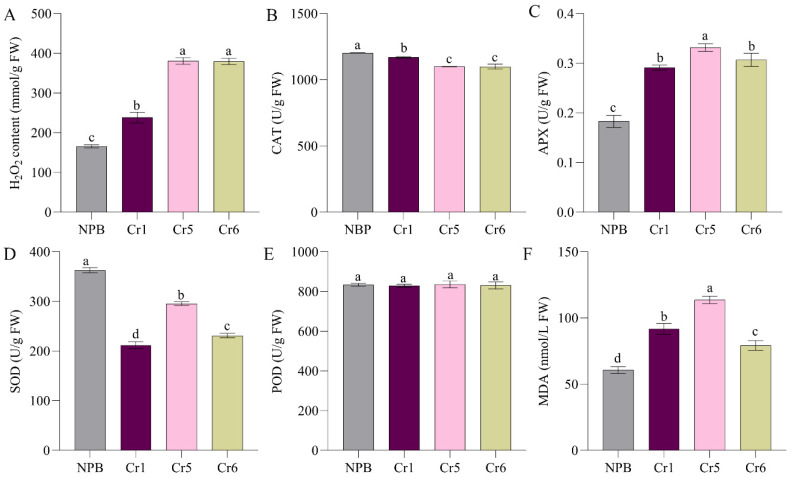
Determination of ROS accumulation of knockout lines. (**A**) H_2_O_2_ content. (**B**–**E**) ROS scavenging enzyme activities. (**F**) MDA content. Cr, knockout; values are means ± SD, *n* = 3; and different letters indicate significant differences by one-way ANOVA and Duncan’s test *p* ≤ 0.05.

In the same way, ROS accumulation in the wild-type NPB and corresponding knockout lines was detected. The results (Figure 5A–F) showed that compared with the wild type, the H_2_O_2_, APX, and MDA contents of the knockout lines were significantly higher. Additionally, the CAT and SOD contents were significantly lower in the knockout lines than in the wild type, while the POD contents did not show a significant difference. These results indicate that the loss of gene function following the knockout of *OsSPL42* disrupted the ROS scavenging system, resulting in the accumulation of ROS.

**Figure 6:**
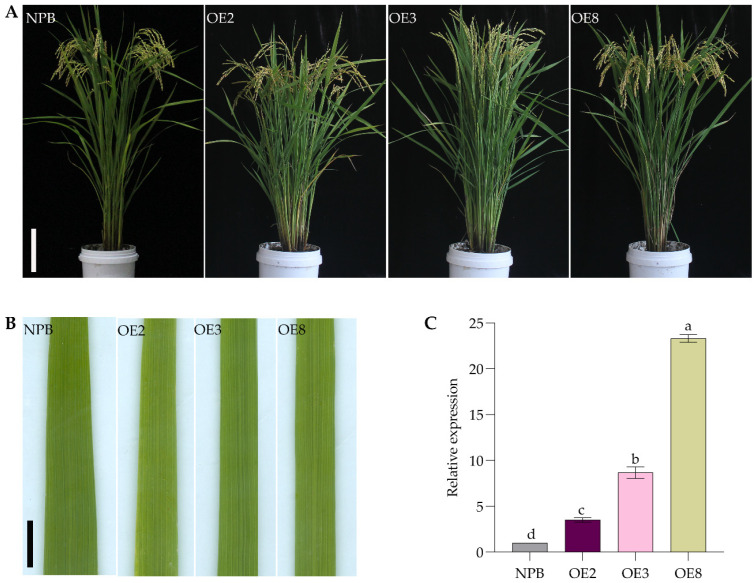
Analysis expression and phenotypes of overexpression lines. (**A**) Plant phenotype of NPB and overexpression line at filling stage. Bar = 17 cm. (**B**) Leaves of phenotype of NPB and overexpression line at filling stage. Bar = 1 cm. (**C**) Expression of *OsSPL42* gene in leaves of wild-type NPB and overexpressed strains at filling stage. OE, overexpression; values are means ± SD, *n* = 3; and different letters indicate significant differences by one-way ANOVA and Duncan’s test *p* ≤ 0.05.

After introducing a binary vector pCAMBIA1300-UBI-OsSPL42, in which *OsSPL42* was driven by a maize UBI promoter, into the callus of NPB, a total of 9 *OE-OsSPL42* overexpressing lines were obtained. The leaves of the overexpression plants exhibited normal green leaf phenotypes like those of wild-type NPB (Figure 6A,B). Expression lines OE2, OE3, and OE8 were selected to investigate *OsSPL42* gene expression, as shown in the figure. The gene expressions of OE2, OE3, and OE8 were significantly higher than those of the wild type by 3.5 times, 8.6 times, and 23.3 times, respectively (Figure 6C).

**Figure 7:**
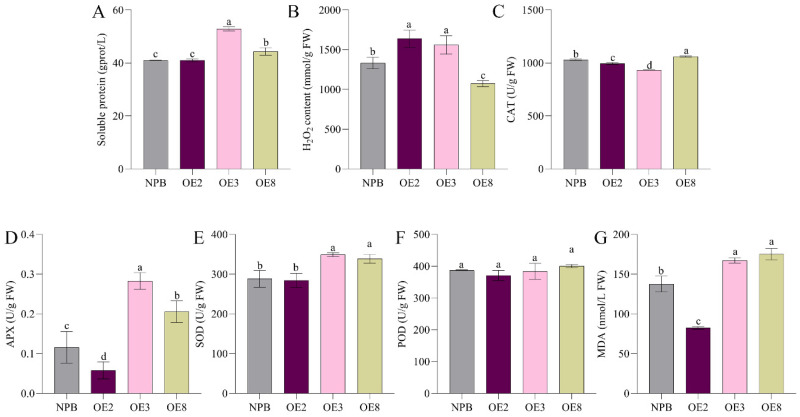
Determination of ROS accumulation of overexpression lines. (**A**) Soluble protein. (**B**) H_2_O_2_ content. (**C**–**F**) ROS scavenging enzyme activities. (**G**) MDA content. OE, overexpression; values are means ± SD, *n* = 3; and different letters indicate significant differences by one-way ANOVA and Duncan’s test *p* ≤ 0.05.

The analysis of soluble protein content and ROS accumulation in wild-type and overexpression lines is shown in Figure 7. The overexpression lines with higher expression levels, OE3 and OE8, exhibited significantly elevated total protein content compared to the wild-type NPB, whereas OE2, an overexpression line with lower expression levels, did not show a significant difference from NPB. The ROS accumulation patterns varied across the lines as well; compared to NPB, CAT activity was significantly reduced in OE2 and OE3, while OE8 showed a marked increase. H_2_O_2_ content in the three overexpression lines showed trends different to those of CAT activity: OE2 and OE3 displayed significantly higher H_2_O_2_ content than NPB, whereas OE8 exhibited a significant reduction. There were no significant differences in POD content among the lines; however, trends in APX, SOD, and MDA content paralleled those of soluble protein. Specifically, the overexpression lines OE3 and OE8 had significantly elevated levels compared to NPB, while OE2 showed no significant difference. These findings suggest that variations in the expression levels of the *OsSPL42* gene influence soluble protein content and ROS accumulation, indicating a potential regulatory role for *OsSPL42* in maintaining ROS balance and soluble protein content in rice. Moreover, these differences in gene expression appear to affect the activities of both oxidative and scavenging enzymes within the plant’s redox enzyme system in a dose-dependent manner.

### 2.4. Enhanced Disease Resistance in Mutation spl42

Spotted-leaf mutants typically exhibit increased resistance to pathogens by inducing HR. In this study, 16 strains of *Xoo* were inoculated with the mutant *spl42*, ZJ100, and a susceptible control variety, Jinggang 30 (JG30), to assess their resistance to BB. The inoculation period occurred during the full tillering stage, and the lesion length was measured using the leaf clipping method 2 weeks after inoculation (Table S2). The disease responses of *spl24* to different strains varied significantly. Among them, resistance to race OS-225 was highly enhanced (*p* ≤ 0.01). Therefore, OS-225 was chosen for the next experiment.

**Figure 8:**
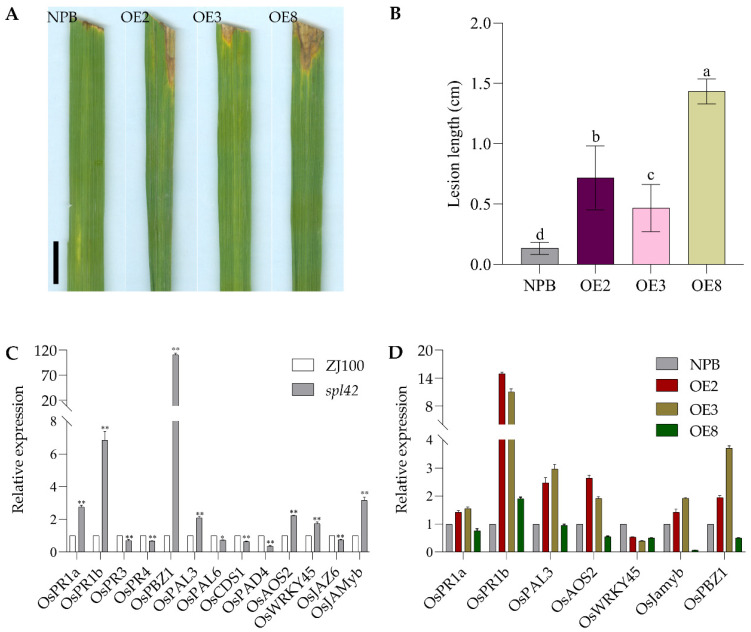
Evaluation of disease resistance in overexpressed lines and analysis of defense response gene expression. (**A**) Results of inoculation of wild-type NPB and overexpression lines with BB pathogen race Os-225 at the tillering stage. Bar = 1 cm. (**B**) Lesion length; values are means ± SD, *n* = 6; and different letters indicate significant differences by one-way ANOVA and Duncan’s test *p* ≤ 0.05. (**C**) Analysis of defense response gene expression of wild-type ZJ100 and mutant *spl42*; the ordinate indicates that ZJ100 is set to 1, and the remaining gene expressions are multiples of ZJ100; *, significance at *p* ≤ 0.05; **, high significance at *p* ≤ 0.01. (**D**) Analysis of defense response gene expression of wild-type NPB and overexpressed strains at the tillering stage. The ordinate indicates that NPB is set to 1, and the remaining gene expressions are multiples of NPB; OE, overexpression.

The resistance of rice mutants to BB is usually associated with the expression of defense response genes. Therefore, the relative expression levels of the defense response gene in ZJ100 and mutant *spl42* were detected. As shown in Figure 8C, compared with ZJ100, the expression levels of 7 out of the 13 defense response genes (*OsPR1a*, *OsPR1b*, *OsPAL3*, *OsAOS2*, *OsWRKY45*, *OsJamyb*, and *OsPBZ1*) were significantly upregulated in the mutant, while the expression levels of the other 6 defense response genes were significantly downregulated. This result is consistent with both increased and decreased resistance in resistance evaluation experiments.

To verify the impact of overexpression on BB resistance, OS-225 was selected to inoculate both the wild-type NPB and the overexpressed plants. The results showed that the lesion length of overexpressed plants was significantly longer than that of the wild-type NPB (Figure 8A,B), indicating that plant resistance was significantly weakened after overexpressing the *OsSPL42* gene. Meanwhile, seven defense response genes (*OsPR1a*, *OsPR1b*, *OsPAL3*, *OsAOS2*, *OsWRKY45*, *OsJamyb*, and *OsPBZ1*), whose expression levels were upregulated in the mutant, were selected for expression analysis, as shown in Figure 8C,D. Overexpression of the *OsSPL42* gene resulted in the dysregulated expression of various defense response genes. The relative expression levels of different genes were either upregulated or downregulated, and the expression levels varied significantly. Compared with the wild type, the expression of the *OsWRKY45* defense response gene was significantly downregulated in the overexpressed plants, while the expression of the *OsPR1b* defense response gene was significantly upregulated in the overexpressed plants. The expression levels of *OsPR1a*, *OsPAL3*, *OsAOS2*, *OsJamyb*, and *OsPBZ1* defense response genes in OE2 and OE3 were upregulated compared with those of the wild type, while those in OE8 were downregulated or showed no change. These results verified that the spotted-leaf mutant *spl42* induced increased resistance to some BB. The results showed that the overexpression plants were weakened to BB, suggesting that *OsSPL42* may negatively regulate the resistance of rice to BB.

### 2.5. OsSPL42 Regulates PCD in Rice

**Figure 9:**
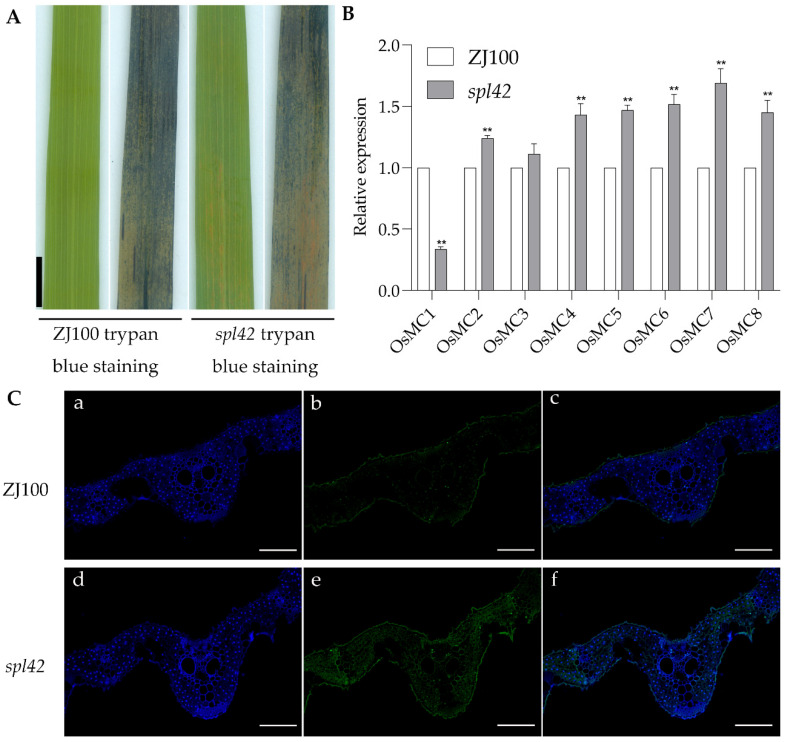
Cell death analysis of the mutant *spl42*. (**A**) Wild-type ZJ100 and mutant *spl42* trypan blue staining at 10 weeks of age. Bar = 1 cm. (**B**) Expression levels of ZJ100 and *spl42* PCD-related genes at 10 weeks of age. Values are means ± SD, *n* = 3; **, high significance at *p* ≤ 0.01. (**C**) ZJ100 (**a**–**c**) and *spl42* (**d**–**f**) TUNEL experiments. The blue signal is DAPI signal, and the green signal is TUNEL signal, indicating the presence of fragmented DNA. Bar = 100 µm.

To verify whether the appearance of spots was accompanied by cell death, trypan blue staining was performed on ZJ100 and mutant *spl42* leaves at the late tillering stage. The results (Figure 9A) showed that blue precipitates appeared in both ZJ100 and mutant *spl42* leaves. However, fewer blue precipitates appeared in ZJ100 leaves, while more blue precipitates were observed in/around the lesions of the mutant *spl42* leaves. Therefore, we used the TUNEL (terminal-deoxynucleoitidyl transferase) method to detect the degree of DNA fragmentation, an apoptosis indicator (Figure 9C). The results showed that more DNA fragmentation (green, TUNEL-positive signals) was detected in mutant *spl42*, compared to that in wild-type ZJ100. This suggests that the *spl42* mutation induced large-scale DNA fragmentation in the cells. Therefore, we examined the relative expression levels of metacaspase (MC) family-related genes, cell death markers [29] in leaves of ZJ100 and mutant *spl42*. The results showed (Figure 9B) that compared with ZJ100, the relative expression levels of *OsMC2*, *OsMC4*, *OsMC5*, *OsMC6*, *OsMC7*, and *OsMC8* in the mutant *spl42* were upregulated and reached a statistically significant level. The relative expression level of *OsMC3* was upregulated but did not reach a statistically significant level. The relative expression level of *OsMC1* was significantly downregulated (*p* < 0.01). In summary, the experimental results here indicate that cell death occurs in the mutant *spl42*, and the expression of related genes involved in the regulation of PCD is altered.

### 2.6. OsSPL42 Is Widely Expressed and OsSPL42 Localizes to Cytoplasm

**Figure 10:**
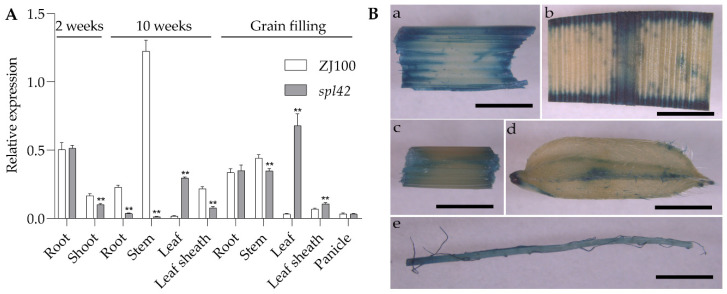
qRT-PCR and GUS analysis of *OsSPL42*. (**A**) Analysis of *OsSPL42* gene expression in wild-type ZJ100 and mutant *spl42*; **, high significance at *p* ≤ 0.01. (**B**) Transgenic plants GUS staining analysis: (**Ba**) leaf sheath; (**Bb**) leaves; (**Bc**) third stem of the plant; (**Bd**) seed; (**Be**) young roots; Bar = 1 mm.

To study the expression pattern of the *OsSPL42* gene, we observed its expression in transgenic plant tissues using the GUS report system, where the GUS gene is under the control of the *OsSPL42* promoter. The results showed that blue color was present in the root, stem, leaf, sheath, and grain of transgenic plants (Figure 10B), indicating that the *OsSPL42* gene was expressed in these tissues. At the same time, the relative expression level of the *OsSPL42* gene in various tissues was also detected by qRT-PCR (Figure 10A). The results indicated that wild-type ZJ100 and mutant *spl42* plants exhibited differential gene expression patterns in the *OsSPL42* gene. Notably, in ZJ100, the expression of the *OsSPL42* gene peaked in stems at 10 weeks after germination. In the *spl42* mutant, the *Osspl42* gene was expressed at the highest level in the leaves at the filling stage. These results confirm that the *OsSPL42* is a widely expressed gene.

**Figure 11:**
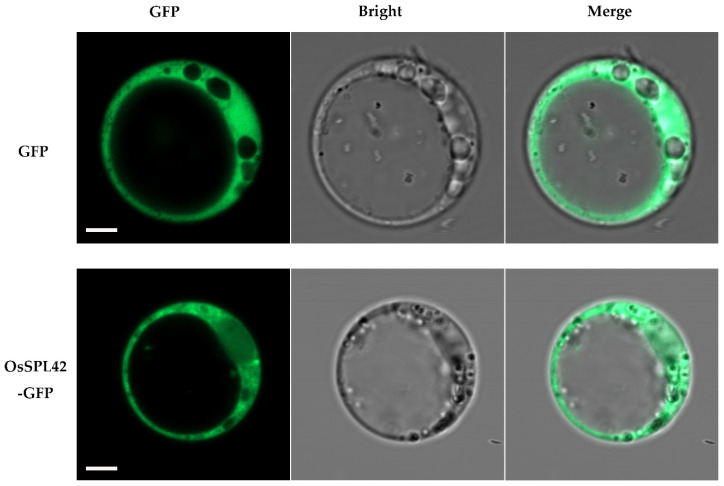
Sub-cellular localization of OsSPL42 protein. Note: GFP, green fluorescence protein; bar = 5 µm.

In this study, the full-length CDS of the *OsSPL42* gene was cloned into the GFP transient expression vector pAN580. The resulting PAN580-*OsSPL42* plasmid was used to identify the intracellular localization of the OsSPL42 protein. Rice protoplast transient transformation technology was used to observe the subcellular localization of the OsSPL42-GFP protein. The results showed that the green fluorescence signal of the OsSPL42-GFP protein was mainly located in the cytoplasm, the same as the signal of the GFP protein alone. This indicates that both OsSPL42 and GFP proteins were mainly present in the rice cytoplasm (Figure 11).

## 3. Discussion

The spotted-leaf mutants exhibit leaf lesions, usually a type of PCD induced by pathogen invasion [30]. These mutants are crucial for the investigation of the molecular mechanisms underlying PCD. Studies have shown that the development of lesions in these mutants often results in enhanced disease resistance, making them ideal subjects for studying the molecular mechanisms of disease resistance [31,32,33]. In this study, the mutant *spl42*, derived from EMS in indica rice, exhibits growth and development deficiencies like other spotted-leaf mutants. By 10 weeks of age, the leaf lesions in mutant *spl42* increase, accompanied by chlorophyll degradation and a decrease in soluble protein content. It is hypothesized that the formation of mutant leaf spots is related to chloroplasts. Many spotted-leaf mutants show a decrease in photosynthetic pigments, which is accompanied by changes in agronomic traits [24,34,35]. In this study, the main physiological and agronomic characters, such as the seed setting rate and 1000-grain weight of mutant *spl42*, were changed by photosynthetic pigment. Many leaf lesions are caused by light exposure [36,37]. The mutants *Spl24* and *HM47* showed light-induced lesion formation under natural light [24,38]. The experiment revealed no significant difference between ZJ100 and mutant *spl42* before and after shading, and spots still appeared in the shaded part of the mutant *spl42* leaves after shading. This suggests that the generation of mutant spots is not light-induced, and thus, the shading experiment is not mentioned in the paper.

In the trypan blue staining experiment, the blue color of the mutant *spl42* leaves was darker, and PCD detection found a large amount of fragmented DNA in mutant *spl42*, with upregulated expression levels of 6 MC family-related genes. The results of these three experiments confirmed the existence of cell death in mutant *spl42*, which may lead to chlorophyll degradation. ROS production is closely related to localized cell death [39]. ROS, a product of REDOX reactions, is removed by corresponding reactive oxygen scavenging systems, including CAT, SOD, and POD, to prevent the harmful effects on cells [40]. The accumulation of ROS in mutant *spl42*, particularly the accumulation of O_2_^−^ and H_2_O_2_, may lead to the death and browning of leaf cells. The SOD activity of mutant *spl42* decreased, while the APX activity increased, suggesting that the damage to the ROS scavenging system might eventually lead to the generation of mutant *spl42* leaf spots.

Lignin can improve plant stress resistance, affecting metabolic pathways [41], salt tolerance [42], and drought tolerance of plants [43]. The COMT gene family can participate in plant abiotic stress responses [44]. In this study, genetic and localization analysis revealed that the mutant *spl42* phenotype was controlled by a recessive gene, *Osspl42*, which encodes an O-methyltransferase protein involved in lignin monomer biosynthesis [45], may be widely involved in various abiotic stress responses [46]. However, the involvement of *OsSPL42* in invasion, such as BB, requires further investigation. *OsSPL42* was found to be the same gene as *OsCOMT*, *OsCAldOMT1*, and *OsCAD2*, encoding caffeic acid 3-O-methyltransferase [28,45,47]. Rice caffeic acid *O*-methyltransferase (*OsCOMT1*) can catalyze the 5-*O*methylation of 5-hydroxyferulate (5-HFA) and 5-hydroxyconiferaldehyde (5-HCAld). *OsCOMT1* can act as *5-HCAId OMT (OsCAIdOMT1)* in the S-lignin biosynthesis pathway and participate in lignin biosynthesis [48]. Lam (2019) showed that the lignin monomers involved in the synthesis of OsCAIdOMT1 include S-lignin, tricin-lignin, and selgin-lignin, with substrates including 5-hydroxyferulate (5-HFA) and 5-hydroxyconiferaldehyde (5-HCAld). The determination of lignin monomer content in rice is more complicated and requires chemical determination. We detected the lignin content of each rice line (Figure S1). The results showed that the total lignin content of the mutant was significantly higher than that of the wild-type and complementary plants (Figure S1A), and the total lignin content of the three knockout lines was significantly higher than that of the wild-type NPB. The total lignin content of overexpressed lines with high *OsSPL42* gene expression was significantly higher than that of wild-type NPB (Figure S1B). This suggests that the abnormal expression of *OsSPL42* might be associated with the accumulation of total lignin in rice leaves. The content of S-lignin monomer in the mutant was significantly higher than that of the wild type (Figure S1C), indicating that the abnormal *OsSPL42* gene might be accompanied by an increase in the content of S-lignin monomer, which would increase the total lignin content. However, this does not prove that *OsSPL42* is negatively regulated with lignin biosynthesis. Due to technical problems, we could not directly detect Tricin-lignin monomer content in rice plants. Therefore, lines with abnormal *OsSPL42* gene were associated with increased total lignin and S-lignin monomer content, but tricin-lignin monomer content may be decreased, which requires further research.

Studies have shown that *OsCOMT* can also inhibit the degradation of chlorophyll and chloroplasts, improve photosynthetic efficiency, and significantly delay the senescence of leaves at the filling stage. *OsCOMT* also positively regulates vascular bundle development [49]. Therefore, *OsCOMT* can improve rice yield through the dual regulation of leaf senescence and vascular development, and is a positive regulator of grain yield, significantly affecting plant height, panicle length, number of filled grains/panicle, and 1000-grain weight [23]. This study confirmed that *OsSPL42* regulates the formation of rice leaf spots, and whether lignin is related to the formation of rice leaf spots is very valuable for further study.

In the protein structure of OsSPL42, the domain of 132–366AA includes a series of *O*-methyltransferases, some of which utilize S-adenosylmethionine as a substrate. This is further evidence that *OsSPL42* encodes an *O*-methyltransferase. The mutant *spl42* was mutated at the 42nd base of *OsSPL42* gene, so the AAs of the 31–89AA domain were changed, but SMART predicted that the dimerization activity of the protein mediated by this structure did not show significant changes. Whether there is any effect on *O*-methyltransferase activity is worth researching further in the future.

ROS can function as a regulator of cell death [50]. DAB and NBT staining showed the accumulation of ROS like H_2_O_2_ and O_2_^−^ in mutant *spl42*. Functional complementation experiments demonstrated that ROS accumulation was absent in the complementary plants, confirming that the mutant phenotype was restored after gene functional complementation, and the ROS clearance system was also restored. This further suggests that *OsSPL42* leads to cell death and regulates the production of leaf lesions in mutant *spl42*, influencing rice agronomic traits. Many genes in plants exhibit pleiotropic effects [51], and traits controlled by multiple genes or quantitative trait loci (QTLs), such as 1000-grain weight [52], can be influenced by other genes or QTLs. In the knockout experiment of the study, only Cr1 showed leaf lesions, while Cr5 and Cr6 did not leaf lesions. We performed off-target verification for the knockout experiments, confirming that off-target effects on other genes were excluded. So, we guess that other genes or QTLs may have compensatory effects on this trait, requiring further research.

The spotted-leaf mutants often enhance plant resistance to pathogens through hypersensitivity, which is an ideal material for studying hypersensitivity and PCD. In the early stage of this study, the resistance of the mutant *spl42* to BB was verified through inoculation with wild type ZJ100, mutant *spl42*, and a susceptible variety (JG30) of rice, *Xanthomonas oryzae* pv. *oryzae*. The results showed that the resistance of the mutant *spl42* to BB was different (Table S2). The resistance of OS-225 subspecies with significantly enhanced resistance was identified on overexpressed plants, and the results confirmed that overexpressed plants had reduced resistance to BB. Therefore, we speculated that *OsSPL42* gene may negatively regulate rice resistance to BB. This was also confirmed by the expression of defense response genes in ZJ100, *spl42*, and overexpressed plants.

The study raises an interesting question regarding the tradeoffs between enhanced BB resistance and other agronomic traits in rice, particularly in the context of *OsSPL42* regulation. *spl42* demonstrated heightened resistance to *Xoo*, with the overexpression of *OsSPL42* in transgenic lines leading to reduced BB resistance. However, *spl42* exhibited reduced agronomic performance and seed quality. Interestingly, these overexpressed lines exhibited improved agronomic traits, such as an increased tiller number (Figure 6). This suggests a potential tradeoff between disease resistance and growth [53]. The reduction in BB resistance in overexpressed plants could be attributed to a reallocation of resources from defense mechanisms to growth-promoting pathways, such as those involving auxins or gibberellins, which are often upregulated in response to overexpression of certain regulatory genes [54]. It is well established that plant immune responses, particularly hypersensitive responses and programmed cell death, are metabolically costly and can hinder growth and yield under certain conditions [55]. As such, the overexpression of *OsSPL42* may prioritize growth at the expense of immune defense, leading to an increase in agronomic performance but a corresponding decrease in disease resistance. To determine the practical implications of these tradeoffs, it will be necessary to conduct field trials that assess both disease resistance and yield-related traits under varying environmental conditions and pathogen pressures. Understanding the balance between growth and defense will be critical for optimizing the use of *OsSPL42* in breeding programs. But future research should also explore the molecular mechanisms underlying these tradeoffs, particularly the signaling pathways and transcription factors involved in the dual regulation of growth and defense. However, the underlying mechanism of this phenomenon requires further investigation.

## 4. Materials and Methods

### 4.1. Plant Materials

*spl42* was obtained from an ethyl methanesulfonate (EMS)-induced mutant bank of ZJ100. The F_1_ population was obtained by crossing *spl42* with a japonica rice, and F_2_ from F_1_ was used for mapping and genetic analysis. The transgenic plants of functional complementation, gene knockout, overexpression, and GUS staining were the experimental materials for functional study, including T_0_ plants and T_1_ and T_2_ plants obtained from self-cross.

### 4.2. Growth Conditions and Agronomic Trait Evaluation

ZJ100, *spl42*, F_1_, and F_2_ populations were grown in the paddy field of the China Rice Research Institute in Hangzhou, Zhejiang Province. The transgenic plants were grown in glass greenhouses at the China National Rice Research Institute (CNRRI), Hangzhou, China. The row spacing of all plants was 16.7 cm × 26.7 cm, with normal fertilizer and water management. After ripening, 3 plants were randomly selected to evaluate their agronomic traits, including plant height, panicle length, number of tillers/plants, number of filled grains/panicle, seed setting rate, and 1000-grain weight. The means from three replicates were subjected to one-way analysis of variance and Duncan’s multiple test (*p* ≤ 0.05).

### 4.3. Determination of Photosynthetic Pigments

First, 0.1 g of fresh leaves were selected from the same part of the rice plants at about 10 weeks of age, and three plants were randomly selected from each group (excluding side-row plants). Then, the method of Arnon and Wellburn was used to determine the photosynthetic pigment content of rice plant leaves [56,57]. We repeated 2 times in the experiment and chose 1 time result to present in the paper.

### 4.4. Histochemical Analysis and PCD Detection

The leaves of ZJ100 and *spl42* at about 10 weeks of age were selected for histochemical staining, including DAB staining to detect the H_2_O_2_ content, NBT staining to detect the O_2_^−^ content, and trypan blue staining to confirm cell death [58,59]. The test of PCD was carried out with the kit provided by using a Fluorescein In Situ Cell Death Detection Kit following the manufacturer’s instructions (Roche, Basel, Switzerland). The principle is terminal-deoxynucleotidyl transferase-mediated nick end labeling (TUNEL). It is a method that can detect the degree of DNA strand break in the process of cell death by fluorescence microscopy. We repeated 2 times in the GUS, DAB, and NBT staining, and chose 1 time result to present in the paper.

### 4.5. Determination of ROS-Related Parameters and Lignin Content

At 10 weeks of age, fresh leaves were randomly selected from three rice plants. The H_2_O_2_ content, soluble proteins content and MDA content, as well as the activities of APX, CAT, SOD, and POD were determined by employing the respective assay kit (Nanjing Jiancheng Bioengineering Institute, Nanjing, China), following the manufacturer’s instructions. Three biological replicates were performed for all measurements. To determine total lignin content, fresh leaves from each sample were dried at 105 °C for 15 min and at 80 °C for 3 days, and then 0.01 g of powder was added to the next test with a kit from Suzhou Mengxi Bio-Pharmaceutical Technology Co., Ltd. (Suzhou, China). Fresh leaves of 10-week-old rice plants were transferred into a biological company (Suzhou Mengxi Bio-Pharmaceutical Technology Co., Ltd., Suzhou, China) using liquid chromatography for the determination of S-lignin. We repeated 3 times in ROS detection and chose 1 time result to present in the paper.

### 4.6. Disease Evaluation

To detect the resistance of the *spl42* to BB, the species of BB were inoculated with the leaf clipping method. Fourteen days after inoculation, the damage length of the inoculated leaves was measured with a transparent plastic ruler. The average of 6 replicates was used for analysis. The BB subspecies were 16 *Xoo*. They were selected from common subspecies in China and representative subspecies in the Philippines including PXO99, GD1385, NX42, Zhe173, OS-225, PXO71, PXO341, HBl7, PXO86, PX079, PXO112, PXO145, PXO280, PXO339, JS97-2, and LN57 (Table S2).

### 4.7. Genetic Analysis and Mapping of OsSPL42

In this study, we constructed the F_2_ population, from which about 500 plants were selected for genetic analysis and 152 plants for mapping. The DNA was extracted from the leaves with lesion mimic using the cetyltrimethylammonium bromide (CTAB) method [60]. We used about 200 pairs of SSR markers linked to the target genes for initial localization and selected 7 pairs of InDel markers for fine localization. Finally, the candidate genes were determined by comparing the base differences in the target interval between the wild type and the mutant by whole genome sequencing.

### 4.8. RNA Extraction and qRT-PCR

The NucleoZOL kit was used for the total RNA extraction. For specific steps, refer to the manufacturer’s instructions (MACHEREY-NAGEL, Düren, Germany). Then, the RNA reverse transcription and qRT-PCR were performed by a kit (Promega kit, Madison, WI, USA). The real-time fluorescent quantitative PCR was performed using a kit ((Thermo Fisher Scientific, Waltham, MA, USA). For specific operation methods, refer to the instructions. The results of qRT-PCR were calculated according to the formula 2^−ΔCT^, 2^−(CT sample−CT Actin)^, with three biological replicates. We repeated 2 times in qRT-PCR experiment and chose 1 time result to present in the paper.

### 4.9. Vector Construction

The infusion method was used in all vector constructions, and the primers used were shown in Table S3. The CDS full-length of *OsSPL42* including 1107 bp was cloned into pAN580, and the GFP vector PAN580-OsSPL42 was cloned into rice protoplasts. The fluorescence signal of GFP was detected by a confocal laser scanning microscope (Carl Zeiss, Inc., Jena, Germany). The GUS transient expression vector was constructed by cloning the promoter region of the *OsSPL42* gene with about 2.6 kb onto pCAMBIA1381Z vector. Then, we introduced the recombinant vector into the callus of the embryogenic call induced from the NPB mature seeds. Transgenic plants were stained using the GUS staining kit (COOLABER SCIENCE & TECHNOLOGY Co., Ltd., Beijing, China). In the complementary validation experiment, PrimerSTAR Max DNA Polymerase was used to expand the full length of *OsSPL42* and part of the promoter and region terminator sequence. The full length of *OsSPL42* was 3168 bp, and the upstream promoter region was 2710 bp. The downstream terminator region is 1518 bp, and the total sequence is about 7 kb. It was then cloned into the pCAMBIA1300 vector, constructed into the PCAMBIA1300-OsSPL42 recombinant vector, and transferred into the mutant *spl42* callus by the *Agrobacterium tumefaciens*-mediated method [61]. We amplified the full length of the CDS sequence of the NPB into the pHY-UBIZ vector to form the pHY-UBI-OsSPL42 recombinant and transferred the recombinant plasmid into the callus of the NPB using the *Agrobacterium tumefaciens*-mediated method. The CRISPR/Cas9 knockout vector of *OsSPL42* was constructed using the NPB as the receptor material and U3 as the vector. The method was mainly based on Liu Yaoguang’s laboratory method system of South China Agricultural University [62].

### 4.10. Sequence Analysis and Off-Target Verification of CRISPR/Cas9 Knockout Targets

It is necessary to carry out the sequence analysis and off-target verification of the knockout target of transgenic plants. The primers used for off-target verification are shown in Table S4. A small amount of the leaf genomic DNA of transgenic plants was extracted, and primers containing 200–300 bp fragments, including knockout targets, were designed. Then, the sequence was amplified and sequenced to verify the success of knockout, and the knockout site of each transgenic plant was analyzed. The target sequence can be found on the CRISPR-P website (http://cbi.hzau.edu.cn/cgi-bin/CRISPR, accessed on 19 May 2021) when searching for homologous sequences in other locations of the target sequence in rice. Primers containing 200–300 bp fragments, including a homologous sequence of the knockout target, were designed, and the sequence was amplified in transgenic plants to verify whether the knockout target was off target.

### 4.11. Phylogenetic Analysis of OsSPL42

The basic information of *OsSPL42* was found in the Rice Database (https://www.ricedata.cn/, accessed on 2 April 2021). We found the domain of *OsSPL42* gene prediction on the SMART website (http://smart.embl-heidelberg.de/, accessed on 18 November 2022). We found the homologous proteins of *OsSPL42* in other species using the NCBI database (http://blast.ncbi.nlm.nih.gov/Blast.cgi, accessed on 18 November 2022). We selected three dicotyledonous plants, *Arabidopsis thaliana*, *Nicotiana tabacum*, and *Glycine max*, and four monocotyledonous plants, *Zizania palustris*, *Zea mays*, *Sorghum bicolor*, and *Triticum aestivum*. These homologous protein sequences were analyzed and compared using DNAMAN v7 (http://www.lynnon.com/ accessed on 18 November 2022). Finally, we used MEGA v6 software (http://www.megasoftware.net/ accessed on 18 November 2022) to construct a phylogenetic tree based on the neighbor-joining method.

## Figures and Tables

**Table 1:** Agronomic traits of wild type ZJ100, mutant *spl42*, and complementary lines.

Material	Plant Height (cm)	No. Tiller/Plant	Panicle Length (cm)	No. Filled Grain/Panicle	Seed Setting (%)	1000-Grain Weight (g)
ZI100	98.0 ± 0.9 ^b^	12.3 ± 1.5 ^b^	23.2 ± 0.6 ^b^	135.8 ± 7.7 ^a^	79.8 ± 0.2 ^a^	19.8 ± 0.3 ^a^
*spl42*	100.9 ± 1.9 ^b^	11.0 ± 1.0 ^b^	24.4 ± 0.6 ^ab^	104.9 ± 9.0 ^b^	60.2 ± 1.5 ^b^	18.2 ± 0.4 ^b^
CP1	98.2 ± 1.1 ^b^	15.3 ± 0.6 ^a^	23.7 ± 0.7 ^b^	130.1 ± 8.6 ^a^	77.7 ± 1.7 ^a^	17.5 ± 0.4 ^bc^
CP2	98.3 ± 2.5 ^b^	16.3 ± 0.6 ^a^	26.1 ± 1.7 ^a^	131.3 ± 27.0 ^a^	78.5 ± 4.3 ^a^	17.9 ± 0.5 ^bc^
CP3	106.6 ± 2.1 ^a^	15.3 ± 1.1 ^a^	25.0 ± 0.9 ^ab^	132.8 ± 14.9 ^a^	78.8 ± 1.7 ^a^	17.4 ± 0.5 ^c^

Note: CP, Complementation; values are means ± SD, *n* = 3; and different letters indicate significant differences by one-way ANOVA and Duncan’s test *p* ≤ 0.05.

## Data Availability

Data are contained within the article or Supplementary Materials.

## Supplementary Materials

The following supporting information can be downloaded at: https://www.mdpi.com/article/10.3390/ijms252211871/s1.
