# Supplementary material for: A Novel Single Base Mutation in OsSPL42 Leads to the Formation of Leaf Lesions in Rice
Source: Int J Mol Sci. 2024 Nov 5;25(22):11871. doi: 10.3390/ijms252211871 (PMC11594205; doi:10.3390/ijms252211871)
Supplement: Supplementary file 1 [file ijms-25-11871-s001.zip › ijms-3185513-supplementary.pdf]

Table S1 Primers used for *OsSPL42* gene localization in this study

Name	Forward primer (5'-3')	Reverse primer (5'-3')
RM337	GTAGGAAAGGAAGGGCAGAG	CGATAGATAGCTAGATGTGGCC
InDelD803	TATTACCATCAACTGATT	TCCTACAACTGACAAGAG
RM22359	TAGGAATAATCGCTGCATGG	GGCTTGAGAGCGTTTGTAGG
InDelD280	ATCAGGTTTACAAGATTCGTC	CAAATCATTTAGCATTAGGCTT
InDelB7	TGGCGATCTCGTCTATGTTCCT	AAAATGATGGTGATTTGACGTTGGAA
InDelD2	GCCCGCACCATAAGCTC	GGCGAACATTGATTTTAATGCAAC
InDelG8	ATCTGGTTGGTTTGATTCGCTTC	GCCTTTTATGCACCACTTCGT
InDelE7	TCCTCCCTATATCTTCTCGGTA	TGCTCATGCCATATTCGTTT
RM22443	GGGAGGTCCTGGATTTATATGTGC	CATTCACAAAGCACACAACACAGC
RM3231	AACACGAAGACCGGCCTC	CAGGTAGGAGCATGAGAGCC
RM25	GGAAAGAATGATCTTTTCATGG	CTACCATCAAAACCAATGTTC
RM310	CCAAAACATTTAAAATATCATG	GCTTGTTGGTCATTACCATTC
RM547	TTGTCAAGATCATCCTCGTAGC	GTCATTCTGCAACCTGAGATCC
RM22690	GGTAGGGATAATTTCGGGACATACC	TCATACACTTTCCAGGTGCAATCC
RM8243	CTCGTGCAACCATTATATTC	ACCTTAGCTGTCCTGAATTG

Table S2 Resistance detection of mutant *spl42* white leaf blight

Race	Lesion length（cm）		
	ZJ100	*spl42*	JG30
PXO86	1.46±1.28	1.88±1.56	3.83±4.30
PXO79	9.23±2.30	8.93±3.26	11.93±3.23
PXO71	7.58±2.39	5.69±1.29*	22.64±3.11
PXO112	7.21±1.85	9.08±9.05	21.07±5.11
PXO99	7.56±3.67	14.32±4.29**	16.18±7.79
PXO145	10.26±3.81	11.06±2.35	16.17±5.93
PXO280	11.83±13.22	9.56±4.66	13.66±5.68
PXO339	13.81±2.67	17.22±5.75	23.79±7.19
PXO341	7.86±2.60	5.80±1.10*	21.10±5.04
JS97-2	2.03±0.62	2.50±0.96	2.96±0.56
HB17	7.07±2.21	5.10±1.38*	21.52±3.99
NX42	8.02±2.16	5.06±1.20**	19.68±4.50
Zhe173	7.70±2.34	5.29±0.91**	21.07±2.84
GD1385	9.55±2.06	12.73±2.77*	11.35±3.23
LN57	7.67±3.03	6.30±1.94	18.44±4.60
OS-225	6.93±1.79	4.43±1.67**	21.25±4.87

Note: Values are means ± SD, n = 9; *, Significance at $P \leq 0.05$; **, High significance at $P \leq 0.01$.

Table S3 The vector construction primers used in this study

Name	Forward primer (5'-3')	Reverse primer (5'-3')	Purpose
Com-6	TATGACCATGATTACGAATTCTCGCCAATAAACTGTGAGACA	ACGACGGCCAGTGCCAAGCTTACTCACACAGTCAATACCAC	*SPL42* complementation
Kon-1	GGCATGGCGTTCTTGAGCGTCAT	AAACATGACGCTCAAGAACGCCA	*SPL42* knockout
GUS-1	ATGACCATGATTACGAATTCTCGCCAATAAACTGTGAGACA	CGACGGCCAGTGCCAAGCTTTCACCGGCGGCGAGTGAGGT	*SPL42* GUS vector
Opv-3	GTTACTTCTGCACTAGGTACCATGGGTTCTACAGCCGCCGA	GATGGATCCGTCGACCTGCAGCTACTTGGTGAACTCGATGG	*SPL42* overexpression
GFP-1	AGTCCGGAGCTAGCTCTAGAATGGGTTCTACAGCCGCCGA	CCCTTGCTCACCATGGATCCCTTGGTGAACTCGATGGCCC	*SPL42* GFP

Table S4 Primers were used to verify the off-target deletion of *OsSPL42* gene in this study

Name	Gene	Forward primer (5'-3')	Reverse primer (5'-3')
Target-1	LOC_Os11g33300	CGTGCATGTCGAAGAACGG	GGCCTAACTCATCCTACCTCA
Target-2	LOC_Os02g11820	TGTTTCGACGCTTATGTCC	TACATGATTTGCACATGGTC
Target-3	LOC_Os02g03620	AGGCTGCACCTTTAACCAC	CATCGTTCACGTCATTGCT
Target-4	LOC_Os01g10820	CTAGCCTCTTGGACTCGTAGC	ATGACACCCTCGTACTTGACC
Target-5	LOC_Os02g10240	TTGTATCTGTTGAAGGTCTTGCG	TATATCCCCTGCAGCATCACA
Target-6	LOC_Os01g07240	CCGCCGCGCTCTCTCGTCT	TGCTCCTGAACGACAGCGAGT
Target-7	LOC_Os08g37456	AACCTTGTATGTTTACGGAG	CACGTACTGGCATGTTAGCAA
Target-8	LOC_Os12g24930	CTGCTGGTCATATAATCCCAT	TGGTTAATCTCTTGCTCGAC
Target-9	LOC_Os01g45030	CAAGGCTGATAAGATTTTCGAT	CTTGATTCTTAACACGCCACT
Target-10	LOC_Os12g22510	GCTGGTCATACAATCTCATCG	CCTGATTCTTAATGCGCCACT
Target-11	LOC_Os04g18730	TGGCATAATTCTGGGTCTCA	CAACCTCCTGCAAGCGTTC
Target-12	LOC_Os08g22180	ATTGCAAGTGGCATAACTCT	CTTTCATCGTCCTCTCGGTCA
Target-13	LOC_Os04g18330	TGCTGGTCATACAATTCCAT	GAGTCTTTATGCCTTCATTGTCC
Target-14	LOC_Os04g18480	TTGATCTGTTACTTCGGGAAA	CCACTTCTGCCTTAGCTTT
Target-15	LOC_Os05g22750	CTTTGATTTACTACTTCGGGAG	CACTTAAGCAACACCCGAT
Target-16	LOC_Os01g28000	CTTTGATTTGCTACTCCGAGA	TGGCTTCGTTTTCTTTAGCTG
Target-17	LOC_Os04g22630	GCTGGTCATACAATTCCATCG	CTTGATTCTTAACGCGCCACT
Target-18	LOC_Os04g16400	AGGCAAAATAAAGTTTGACGA	ACTTGATTCTTAACACGCCAT
Target-19	LOC_Os03g39500	CTTTTCCTGTCAATATGGTGC	TTCGTCTTCTTTAACCGAT
Target-20	LOC_Os01g22850	CTGCTGGTCATACAATCCC	TTCTATTATACGATCGGCTT

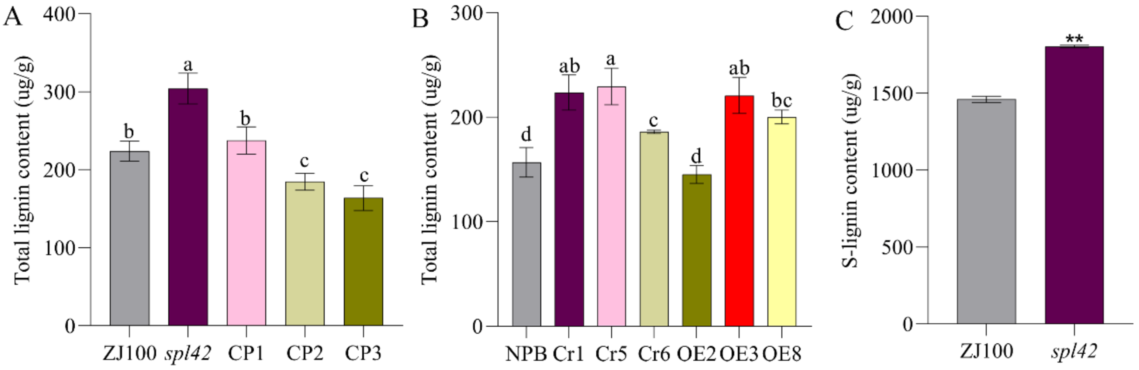

Figure S1 Detection of lignin content in mutant, wild type and transgenic lines
(A) Total lignin content of wild type ZJ100 and mutant *spl42* and complementary lines at tillering stage; (B) Wild-type NPB and knockout and overexpression lines of total lignin contain; CP, Complementation; Cr, Knockout; OE, Overexpression; Values are means ± SD, n = 3; and different letters indicate significant differences by one-way ANOVA and Duncan's test $P \leq 0.05$; (C) Contents of syringal (S-lignin) in wild type ZJ100 and mutant *spl42* at tillering stage; Values are means ± SD, n = 3; *, Significance at $P \leq 0.05$; **, High significance at $P \leq 0.01$.
